# “That Was the Happiest Time of My Life”: Understanding Childhood Eco-Connections in Appalachian Communities

**DOI:** 10.3390/ijerph192416661

**Published:** 2022-12-11

**Authors:** Sherry Hamby, Katherine M. Montgomery, Heather L. Storer, Victoria Banyard

**Affiliations:** 1 Department of Psychology, The University of the South, Sewanee, TN 37383, USA; 2 Life Paths Research Center, Sewanee, TN 37375, USA; 3 Kent School of Social Work, University of Louisville, Louisville, KY 40292, USA; 4 School of Social Work, Rutgers, The State University of New Jersey, New Brunswick, NJ 08901, USA

**Keywords:** ecopsychology, connection to nature, sense of place, place attachment, Appalachia

## Abstract

The goal of this study was to explore facets of childhood eco-connections, using retrospective qualitative data from adults. One hundred and forty-five adults from predominantly rural and small-town communities in southern Appalachia (71.7% female), average age 36.23 years (SD = 12.08) participated in semi-structured qualitative interviews on key life experiences and the places they grew up. Mentions about place-related childhood memories or interactions were grouped into four themes based on exploratory thematic content analysis: nature nostalgia (positive reminiscences about nature experiences); nature immersion (extensive contact with the natural world during childhood); formative experiences with nature (nature interactions that taught a skill or life lesson); and rhythms of nature (appreciation of seasonal or cyclical patterns). Childhood eco-connections are multifaceted and often emerge from early impactful or intense experiences. Pro-environmentalism messages to youth may benefit from more references to childhood eco-connections.

## 1. Introduction

Connections to nature benefit children and youth in a multitude of ways. Developmentally, spending time in nature in childhood has been associated with lifelong engagement in environmental stewardship [1,2,3] and preference for outdoor activities in adulthood [4]. Using a lifecourse perspective, Wells and Leikes [5] documented that early experiences in non-domesticated or “wild” nature were critical for forging a pathway towards a lifelong commitment to caring for and enjoyment of the natural world.

In addition to strengthening children’s bonds to nature, immersion in nature is also an unexamined determinant of physical and mental health. The presence of green space during childhood, for example, is associated with lower risk for mental health problems [6] including alleviating stress, reducing cognitive and sensory overload, and nurturing feelings of well-being [7]. Meredith and colleagues [8] reported that spending 10 min in nature a day is an adequate “dose” to positively impact college students’ mental health. Furthermore, exposure to nature can promote increased school achievement and cognitive performance [9,10]. There is a large literature exploring connectedness to nature or other facets of *eco-connections*, which are the quality and strength of our ties to the natural world [11,12]. However, as a prior review noted, the field could benefit from research that creates meaning regarding the various dimensions of eco-connections [13]. The purpose of the present study is to further the conceptual analysis of connections to nature by inductively exploring different facets of childhood eco-connections as they are remembered by adults in rural Appalachian communities. As has been noted by others [1,5,14], conservation education and investment in environmental causes continues to be a challenge in many regions, and better understanding eco-connections may facilitate environmental education aimed at youth. We are also interested in better understanding eco-connections as a psychosocial strength that can promote well-being across the lifespan.

### 1.1. Eco-Connections and the Psychology of Place

Like many constructs in the social sciences, a variety of related terms have emerged to describe bonds to the environment. *Connectedness to nature* has been defined as people’s subjective sense of their relationships with the natural world and their sense of inclusion in nature [15]. Another common construct is *place attachment*, defined as the emotions and feelings individuals attribute to a location, usually a specific place such as someone’s hometown [16]. Such attachment has theoretically been linked to youth identity development and meaning making about potential life trajectories [17]. *Sense of place* is a multidimensional construct representing emotions, beliefs, and behavioral commitments surrounding a certain geographic setting [18]. These latter two constructs could include natural spaces but also encompass built environments and geographic locations like towns.

Although often discussed separately, in practice there are not always clear boundaries among these concepts, which can be seen in the overlapping ways that they are often operationalized. For example, although place attachment can refer to urban environments, it is sometimes described as a sense of connection and kinship with nature; connectedness to nature is typically defined using very similar language [19]. Associations with nature and interest in nature activities and natural spaces often overlap in definitions of these terms [13,20,21,22].

Numerous studies have examined the healing and restorative properties associated with time spent in nature [23,24]. For example, there is a growing body of literature that measures interactions with specific natural spaces, including *shinrin yoku* (“forest bathing”) that focuses on green spaces, and the impact of water or “blue space” on variables like perceptions of well-being [25,26]. Further, a growing area of study documents the crime-reducing effects of greening urban spaces, a promising intervention that nonetheless could benefit from a deeper understanding of how green spaces create prevention processes [27].

Many quantitative measures of these constructs rely on global assessments of the natural world, as can been seen in a recent compendium of measures [28]. For example, the Connectedness to Nature Scale [29] asks about “a sense of oneness with the natural world around me”. A scale on place attachment asks similar questions, but specific to a single area, such as “When I spend time in the [mountain range], I feel a deep feeling of oneness with the natural environment” [30]. This overlap is not unusual, raising questions about the boundaries of the constructs and the range of experiences that contribute to eco-connections. These are also good examples of the global level at which eco-connections are usually assessed, with many scales, as noted in prior reviews, focusing on measuring overall positive feelings or thoughts about the natural world, time spent in the natural world, or commitment to environmental causes, often combining responses to items into a single score [13,28].

*Eco-connection* is an emerging term that encompasses all these constructs and can describe other adaptive, sustainable relationships to nature (versus exploiting the natural world for resources) [11,12,31]. As Kahn [14] has noted, eco-connections are distinct from affiliations with other humans, the built environment, and other human-created artifacts, although in some sense these are all products of nature as well. It is widely acknowledged that eco-connections are first formed in childhood [14]. Eco-connection has also been suggested as an antidote to the increasing experience of eco-anxiety in this period of climate change [32,33]. Although studies indicate that there are variations in types of eco-connections, more research is needed to contextualize these variations and contribute to a greater understanding of the processes by which eco-connections can nurture individuals’ well-being.

### 1.2. Prior Qualitative Research on Eco-Connections: Strengths and Limitations

Extensive qualitative research has examined the development of relationships with nature during childhood, the benefits of exposure to nature, and the pathways to environmental activism, often focusing on children and youth [14,29,30,31]. Inductive qualitative research offers the potential to elucidate the processes by which nature can strengthen individuals’ and communities’ attachment to the natural environment [32]. Numerous qualitative studies have shed light on the positive psychological aspects of people’s relationships with the natural environment [14,34,35,36,37,38,39]. Some studies are devoted to a particular place or issue such as economic change, local laws, or climate change. Although many of these studies focus on adults [40,41], some have focused on youth, such as the impact of youth’s contact with green spaces in urban environments in the UK [38] and the potentials of beaches for health promotion [34].

Although many of these studies note children’s affinity for natural spaces and the positive elements of eco-connections, there has been less attention to variations in the ways that eco-connections are experienced in childhood, how eco-connections emerge, or how they are understood by adults reflecting on their developmental life history. These are key questions that may help clarify potential links between eco-connections and well-being across the lifespan. This understanding may be particularly important to enhance strengths-based work in under-studied and under-served rural communities who may have greater access to eco-connections. Indeed, studies of green space in disciplines like urban planning raise issues of lack of access or inequitable access to green spaces in urban communities [42] and more deficit risk models [38]. Eco-connections may, by contrast, be an available strength in rural communities (who are also often studied mainly in terms of risk factors and whose resilience needs to be better documented) [43] and studies show migration to rural areas may be driven by greater access to green spaces [44].

### 1.3. The Current Study

This study is informed by the resilience portfolio model [45,46], which identifies the full range of psychosocial strengths at all levels of the social ecology (families, peers, and communities that contribute to individual functioning), especially the often-overlooked strengths of underrepresented communities. In this model, *resilience* is defined as the process of using psychosocial strengths to overcome adversity and achieve wellbeing, with the hope of identifying the key strengths that support resilience [47,48,49]. One goal of the model is to explore under-appreciated facets of popular constructs such as “social support” or “eco-connections” to elucidate how these strengths support thriving [46,47]. The resilience portfolio model is employed in this study to explicate the theoretical pathways explaining the relationship between childhood connections to nature and individual-level well-being and resilience across the life course. This model helps explain the relationship between individual’s meaning-making of sensitizing events and resiliency related outcomes in adulthood. Thus, in the context of this study, the meaning that people attribute to their early lived experiences in nature provides a lens for understanding how these experiences contribute to lifelong positive functioning, place attachment, sense of purpose, and commitment to environmental stewardship. This work makes a positive contribution to the literature because work in environmental psychology, for example, has not been well-integrated into other research on psychosocial strengths, and fields that consider geographic spaces (including landscape architecture or urban planning) are seldom directly connected to studies of human behavior in fields like psychology or public health. Communities are influenced and often even defined by their physical spaces; for this reason, we need to better understand all the ways that eco-connections manifest.

The purpose of the current study is to understand the ways that people talk about their childhood experiences and feelings toward nature, with a particular focus on identifying under-appreciated elements of eco-connections. The findings in this study illuminate how myriad different dimensions of eco-connections contribute to their well-being. The authors conducted a large number of interviews from rural southern Appalachia and surrounding areas, in which participants were asked general questions about key points in their life, including where they grew up. In doing this, nature was offered spontaneously as a sensitizing construct to create meaning regarding their childhood experiences. The goal of this analysis is to identify the types of eco-connections occurring during childhood as described by participants.

## 2. Method

### 2.1. Participants

The community sample included 145 adult participants (71.7% female), ages 21 to 69 years (M = 36.23 years; SD = 12.08 years). Most participants were European American/White (75.5%); 12.6% identified as Black/African American, 4.9% as Asian American/Pacific Islander, 4.9% as multiracial, and 2.1% as Latino/Hispanic (any race). The sample was somewhat more racially and ethnically diverse than U.S. Census data from the area. Participants were recruited from seven counties in southeastern Tennessee and northern Alabama that are designated Appalachian by the Appalachian Regional Commission. The counties are composed of predominantly rural areas (population less than 2500) or nonmetropolitan small towns (population 2500–20,000) and have average incomes that are below the U.S. median household income, according to U.S. Census data. These data are from a larger study on psychosocial strengths and resilience [46].

### 2.2. Procedure

Our study used a combination of convenience and snowball qualitative sampling techniques. Most participants were recruited via community events like county fairs (76%), followed by word of mouth from prior participants (12%), with others recruited by media such as direct mail to households in the county where our research center is located, newspaper and radio ads, and flyers. Participants were invited to take part in an interview on personal strengths, resilience, and key life events. Interviews took place in 2013 and 2014 at locations throughout the community, including our research center, participants’ homes, restaurants, and community events. Individuals who participated in the interviews received a $50 Walmart gift card for their participation.

Using a semi-structured interview guide, interviewers asked participants to discuss their life stories by describing high, low, and turning points, positive and not-so-positive behaviors, past and current challenges, and coping strategies. They were also asked to describe the context of their early childhood. There were no specific questions about connectedness to nature or other eco-connections in the interview, as this was not a focus of the original project. As a result, all mentions of nature or places emerged organically from responses to more general questions about their life, primarily about where they grew up. The interviews were semi-structured and typically lasted approximately an hour. Interviews were audio recorded and transcribed verbatim. The transcripts are available from the authors. All procedures were approved by the home institution’s institutional review board.

### 2.3. Data Analysis

We used a thematic content analysis approach to guide our analytical process. Thematic content analysis is a systematic process of constructing patterns and themes in the data, including noting divergences or outliers [50,51]. This methodological approach is consistent with the inductive nature of our inquiry. We used multiple rounds of qualitative coding including open, axial, and selective approaches [52]. In the “open” phase, the second author and a research assistant evaluated the first 10 transcripts to develop a sense of the types of eco-connections mentioned, focusing on transcripts that included any mention of nature (such as woods, farms, animals, etc.). This iterative process allowed codes to emerge inductively. We did not use a priori codes for several reasons. Not only is this approach not epistemologically consistent with the inductive nature of thematic analysis [53,54], but the study of eco-connections in Appalachia is formative, so there is not an existing theoretical base to inform the development of relevant pre-existing codes. Transcripts mentioning nature were noted as to how nature and the participant’s connection to nature was described and then characterized in more general categories. For example, some early categories were “mentions an annual event related to place,” “learning about nature,” and “grew up in the country”. Virtually all quotes about eco-connections pertained to childhood, so further analyses were limited to experiences in childhood only. In the second “axial” phase, these initial categories were refined, and exemplary quotations were discussed with the first author. The categories were subsequently applied to remaining transcripts, including noting outlying counterexamples. Identifying counterexamples (i.e., negative case analysis) is a critical step in thematic content analysis in order to demarcate the boundaries around a concept by identifying instances that fall outside the theme or category [55,56]. In our analytical process, we note counterexamples to acquire some specificity around the domains of people’s eco-connections. In the third and final “selective” phase, the authors reached a consensus by integrating and combining categories into four core themes.

In the interest of reflexivity, a benchmark of rigor in qualitative studies [57], we wanted to briefly discuss how our personal identities and lived experiences influence our analytical approach. SH has lived in the region where this study was conducted since 2008 and has multigenerational roots in Appalachia and the southern US more broadly. She lives in a small town and spends time each day on the wooded streets and trails of her community, which helps to sustain her wellbeing. Her work focuses on pathways to overcoming trauma. KM is currently an MSW student in Texas and is interested in the natural environment’s effect on individual resilience. She was an intern at the research center where this study was conducted and lived in this community for four years. HS is predominantly a qualitative researcher interested in how technology can support abuse survivors’ well-being. She lives in an exurb surrounded by nature, so appreciates the restorative aspects that nature provides to balance our lives in the digital age. VB uses a range of methods to understand the strengths of individuals and communities that can help prevent interpersonal violence. She lives in an urban area but seeks out natural spaces both within cityscapes and outside of them to cultivate a sense of centeredness.

## 3. Results

Participants’ responses during interview sessions identified various ways that nature was a sensitizing construct in their childhoods. Detailed stories about nature came in response to a question asking them to describe the context of their childhood (sometimes in different parts of the country than where they currently lived). Four themes were constructed to describe four distinct components of participants’ eco-connections: nature nostalgia, nature immersion, formative experiences with nature, and rhythms of nature.

### 3.1. Nature Nostalgia: “That Was the Happiest Time in My Life”

The theme of nature nostalgia encompasses the remembrances participants shared regarding the ways connection to nature punctuated their youths. These stories depict heartfelt memories of time spent in natural environments and represented touchstones of positive emotions in the life narratives of participants. Within this theme, participants used positive words or phrases such as “happiest time of my life” (38-year-old woman), “just good memories” (51-year-old woman), or “highlight of my life” (60-year-old woman) to demonstrate the breadth of their reverence for their childhood experiences in nature.

Participants’ nostalgia involved different aspects of nature, including time spent gardening, caregiving for farm animals, and unstructured time in woods or fields. For example, one 38-year-old woman described the joy of gardening with a neighbor. They stated:

[I] would be over on Mr. [neighbor’s name]’s side of the property. And then he had the blueberries. And the blueberries I remember. Oh, the blueberries! And he had the honeybees, and he had the grapes and all … *He just loved to grow things* [all emphasis added] … We would walk that mile to go to his house … *That was the happiest time of my life.* (38-year-old woman)

In addition, there were mentions about the love of spending time in community with animals and the pleasure of freedom to explore. One participant, a 60-year-old woman, stated, “Riding the horse out in the mountains I think was a *very high point* for me. I lived in [place], and I had a wonderful horse. So those times I think were the highlight of my life”.

This sentiment was share by a 51-year-old woman who recounted:

Life was simple … That’s where *my love of animals* was. I helped my grandfather in his garden and he had goats that he raised, and we had to feed some of them with the coke bottles and a nipple. And I’d help go with him when he had to go swarm some bees, ’cause he was a beekeeper … *just good memories.*

Some participants did not have a childhood full of these moments, but spoke of memorable events in nature that stuck with them into adulthood:

Oh yeah, that time we were in Florida, we was at the beach, and I was scared to death getting in the water, you know, the water looked like it was massive above my head. And [my mother] took me out there, put me on her shoulder and the water just come up, like, she went in for her knees and I was like looking and I’m thinking if I stand up there and I was, like: “Damn, we’re all the way back, my God, we were that far and we’re only that deep?” And I told her: “Put me down.” And the whole time we was out there she held my hand and it just made me feel really good, you know. *That’s definitely my happy memory. Oh, I want to go back to that happy memory*. (37-year-old man)

Not all nature memories were pleasant. A counterexample was provided by a 27-year-old man whose cousin drowned after getting caught in a sinkhole. In addition to the traumatic nature of this experience, it shows the power of forces of nature.

I ain’t getting in that water, I’m scared. So, I go back down to where we came through the woods. So, I’m at the woods and I hear somebody saying, ‘Uh, where’s [cousin’s name],’—that’s his name—said, ‘Where’s [cousin’s name]?’ Said, ‘I don’t know, he ain’t come up yet,’ and I had just saw my cousin come up and I had seen him go and catch his breath and he went back under like, okay, I had seen (cousin name) and I know he’s good so he’s swimming underwater … a couple more seconds go by, I was like, something ain’t right, and then they’re like calling them all at once. So, they called the ambulance, I mean, rescue squads and stuff … Rescue squad came, uh, did what they could do … Said they had got caught in the sinkhole or something and when we walked across to it, the water was probably 4-and-a-half foot at the most … When they had swam up under the water, I guess the current was the one that took them. They both got caught in the sinkhole. It took them about, like I said, about 4 h and about 6 h to get him up out the water and I don’t know, *that was just like a changing point* in my life, ‘cause he was born in ‘86 and I was born in ‘86, I mean it was my cousin.

### 3.2. Nature Immersion: “I Was Outside a Lot.”

Another dimension of eco-connections was illustrated through participants’ ongoing *immersion* into nature. Within this theme, participants described the importance of sustained time spent in contact with the natural world during their childhoods, whether through recreational activities, working in nature, or living in an environment surrounded by nature. Sometimes the extensive contact was discussed in terms of time spent: “It was fun out there [in nature]. There was lake back there, *we fished everyday too*. We still go out there and fish. To this day, still going out there” (27-year-old man). Other times, immersive contact was framed in terms of connection to physical space, as when a 39-year-old woman stated, “It was 33 acres of bayou-front property. The corner property had bayou *on all three sides*. I grew up living with alligators, opossums, and … rattlesnakes, big king rattlesnakes”. In this instance this woman was literally surrounded by the expansiveness of the bayou. Although these statements often had positive connotations, they differ from nature nostalgia in their focus on the ongoing immersive aspect of their extensive contact with nature.

In addition, the below quotes exhibit different experiences of immersion during childhood, all with a positive perspective.

Alabama rooted me in a really good way, ’cause I stayed there the longest and I was doing things that were important to me and riding horses and farm animals and, you know, riding in the back of a pickup truck through fields and feeding cows and *just that sense of open space and freedom* and being around stable people that are in *the same place all the time* and that loved you dearly. (57-year-old woman)

There was a, like, we called it the woods or whatever, but it’s really not that big of a woods, but it was, you know, *a lot of trees, and it had a little creek*. We’d go and, you know, play in the water, and go out there and roll up different kinds of grass and stuff and be trying to smoke it, rolling it up with notebook paper, just being bad, and just running around. *I really enjoyed that part of my childhood, just running around with my sister and my friends in that neighborhood*. (31-year-old woman)

The following quote is a counterexample from a 26-year-old woman who has negative views of some aspects of nature in both her new home and the place in Texas where she lived previously:

Adjusting to the different culture and temperature and everything, of moving from the lake here [in Tennessee] to Texas … I mean it was really different. Now, I was really not very happy with the heat at first. But I definitely acclimated to it. [When I was in Tennessee] I was outside a lot when I was here, and I would run barefoot around my grandmother’s yard because she kept me all the time. And *you can’t do that in Texas because there are fire ants … you can’t just go run around barefoot.* And really the grass doesn’t grow. It was, it was kind of culture shock at first.

### 3.3. Formative Experiences with Nature: “It Really Shaped How I Saw Things”

Across the sample, participants described how their childhood interactions with nature came to represent formative experiences that taught them a lasting skill or life lesson that stayed with them in adulthood. Participants used language such as “it really shaped how I saw things” (22-year-old woman) and “it was, like, a learning experience” (45-year-old woman) to emphasize the enduring and formative impact connections to nature had on them developmentally. Unlike nature nostalgia, in which participants were sentimental about the influence of nature, or nature immersion, which focused on the impact of in-depth and sustained time spent in nature, participants construed formative experiences with nature as helping facilitate a sense of responsibility, confidence, routine, or connection to critical people. As the following passages illustrate:

*The farm was possibly the most influential part of my growing up*. I grew up, you know, working on the farm and it meant that I had a little bit of a different viewpoint when I got to college … *I think that it really shaped how I saw things* because it meant that I was working every day … you just can’t go to school without feeding animals or without weeding a garden because before you know it, it’s gone. So, I really understood hard work because, you know, you always have to go out and … like this morning I was home for a day and a half and my parents had me picking okra and squash, you know. (22-year-old woman)

It, uh, teaches you a lot, you know, of respect of yourself and respect of the land, you know. Because if you don’t take care of it, you can’t put something out there and it grow by itself, you have to take care of it, … and *so it was, like, a learning experience. And I tried to learn what I could*, you know. (45-year-old woman)

Living out there gave me, *like it taught me about nature. It taught me about working hard*. My dad would make us, you know, go out and work with him and stuff like that and mess with the garden and things like that. And so, *it gave me a sense of responsibility*. … So, *it definitely shaped* who I am. Just gave me the confidence. (22-year-old woman)

One counterexample was provided by a 51-year-old woman who found that not all lessons learned during childhood translate to all environments.

… up north [where she grew up] there were sidewalks everywhere, you could ride your bicycles on the sidewalks, ride ‘em all over, seemed like there was more, uh, to do outside, you know what I mean? And in the South, they don’t have that many sidewalks for kids to ride their bikes to be physical and things like that, it’s a little bit more dangerous, ‘cause kids have to ride on the road and I think that’s, uh, quite a bit more dangerous…

### 3.4. Rhythms of Nature: “My Dad Grew a Garden Every Year”

Some participants mentioned seasonal and cyclical occurrences such as planting, farming, slaughtering, seasonal family vacations, and weather patterns as memorable moments in their childhoods. In contrast to the previous themes, these remembrances underscored how the rhythms of the natural world were central to their sustenance and to their relationships. Instead of references to daily activities or responsibilities, as in nature immersion, these comments referred to annual or seasonal occurrences that renewed their connections to their families and communities. In these narratives, nature provided a way to mark time and to signal community events or engagement opportunities. For example, one 60-year-old-woman reflected on how every summer “the family *always took a month vacation*, and we would go up to northern Michigan and we had a, my family had a cabin up there and we *would spend the whole month together as a family*”. A 47-year-old woman noted, “My dad grew a garden every year”. Thus, these annual shared experiences evoked feelings of family connectedness. Similarly, a 60-year-old woman shared how consistent weather patterns initiated increased visiting with community members:

When it rained on this island, that was sort of an understood tradition that that’s when people might come visit you. so when it rained you *just sort of expected to do it.* I, I would go visiting or you would be visited by people, which was kind of fun.

In addition, participants described how the sharing and slaughtering of farm animals came to demark a specific period of time. For example, a 54-year-old man stated, “My dad raised swine and we, that’s how we survived in life, is he raised a big garden, we’d can things to the winter, for the winter…” Another participant commented on how this sharing of resources further affirmed their connection to one another: “You know, it was just a really great community, you know. The people we rented our property from, the farm, they raised cattle on it. *Every time they’d slaughter a cow, they would come fill up our freezer*” (30-year-old man).

## 4. Discussion

Four different aspects of childhood eco-connections were identified in interviews in this rural southern Appalachian region: nature nostalgia, nature immersion, formative experiences with nature, and rhythms of nature. The themes all reference sensitizing or impactful childhood experiences that reaffirmed participants’ connections to their communities and the natural world. Nature nostalgia refers to powerful, emotional, and positive memories of nature experiences. In this theme, connections to the natural world were described as distinct positive life events, perhaps akin to benevolent childhood experiences identified in the resilience literature [58]. Nature immersion represents participants’ descriptions of in-depth exposure, either in terms of long periods of time or being surrounded by nature. In this theme, nature is seen as an important developmental context—part of the participants’ psychological ecosystem. Any experiences where a participant learned a lesson or skill through nature represent formative experiences, in which nature can be seen as teacher or as a driver of development. Rhythms of nature include references to cyclical events such as seasonal changes in growing seasons, harvesting, or weather. Nature was both a timekeeper and a signal for experiences that brought communities together.

Participants’ vernacular regarding their time in nature adds meaningful aspects that quantitative data could not capture. Further, the negative cases also remind us that eco-connections are complex, with some participants having negative connections and even reminders of traumatic loss in nature. These data suggest new directions for considering the theoretical and nuanced aspects of eco-connections during childhood, going beyond global positive feelings about nature or place by suggesting four elements that help comprise those feelings. These data are consistent with previous inductive research that has delved into perceptions regarding a single locale, especially regarding the psychological effects of how the physical environment impacts ways of feeling about oneself and others [34,38]. However, instead of looking at one aspect of a child’s experiences with nature, such as families’ time at the beach or time spent in a nature school, this study, with its open-ended prompts and people living in numerous communities in a seven-county region, allowed participants to share a wider range of nature memories from any place or point in their life. This breadth allowed us to derive themes that can apply to multiple locales while still offering more detail than global positive feelings.

Our results suggest that eco-connections are multi-faceted and that some key factors in developing eco-connections are the intensity and impact of the experiences. This contrasts with many existing quantitative measures, which often ask broad questions about positive thoughts and feelings about nature or break down eco-connections into cognitive thoughts about relationships between people and nature versus emotional connections and are multidimensional [13,59], which may not fit with participants’ subjective lived experiences. Although some participants referenced childhood experiences from outside the region, the emphasis in their comments on intense and impactful experiences may arise in part from living in the Appalachian region, where immersive contact with the natural world is still relatively common. This suggests an area of regional strengths that should be explored in more detail in future research. Indeed, a key next step could be interviews designed more specifically to capture eco-connections, including asking more structured questions about the perceived impacts of these experiences. Such work could set a foundation for quantitative measures to test the connection more specifically between different types of eco-connections and well-being outcomes. Prior research documents, for example, how creating green spaces in urban environments can promote a sense of community and reduce problems like crime [27]. More nuanced measures of eco-connections, such as the dimensions described in the current study, might provide more understanding of the mechanisms that produce positive effects of natural environments.

The counterexamples to these themes help to show the boundaries around these concepts—not everyone is nostalgic about nature, had the opportunity to learn lessons from the natural enough to learn lessons, or feel immersed in it. Further, the counterexamples also show that the natural world can be a source of trauma and pain as well as oneness and awe. There is a large literature on trauma and resilience related to natural disasters [60] and linking this literature to research on eco-connections may help explain individual variations in levels of eco-connection. To date, studies of natural and green spaces are often associated with fields like urban planning, landscape architecture, geography, and criminology. The current study took a more psychological and developmental approach [47]. These counterexamples provide more nuance to our understanding of eco-connections.

## 5. Limitations

This study has limitations that should be acknowledged. Although the sample is large compared to most qualitative research, participants were largely similar in terms of socioeconomic status and race. This is consistent with demographics of the area, but it would be important to explore eco-connections in other communities, especially given the range of historic experiences and relationships that different communities have with nature and ways that access to nature or appropriation of natural spaces have been used as tools of systemic oppression. The data are cross-sectional and longitudinal studies of eco-connections would allow for exploration of changes over time. This was an exploratory analysis for a project that was not focused on eco-connections. Although the strength of this is that it reveals how childhood eco-connections emerge organically in discussion of key life stories, future research would benefit from intentional protocols to assess childhood eco-connections.

## 6. Conclusions

The themes from this study suggest several avenues that can be explored in future research and intervention. Regarding research, the themes identified here can be used to create new measures to further explore the themes and their associations with other relevant variables. As we noted in the introduction, many measures of eco-connections only ask global questions about the degree to which such connections are present, and we are not aware of any that explicitly ask questions about nature nostalgia, nature immersion, formative experiences, or exposure to the rhythms of nature. More generally, we are not aware of any that assess the role of impactful or intense experiences in developing eco-connections. These dimensions may also be useful for understanding a relatively new but related concept, *solastalgia*, which refers to the distress created by climate change, which is increasingly creating intense and impactful experiences [61]. New measures of eco-connections could include subscales for each of these categories—or explore others not yet identified. It would be beneficial to explore dimensions of childhood eco-connections in other communities. For example, other researchers have noted that experiences such as immersion in nature may not be prominent in urban settings or may look quite different than those experienced in more rural areas [36].

Results from this study also suggest potential avenues for pro-environmentalism or conservation messages, which interact in complex ways with geography and place [62] (see for example a discussion of teaching ecological science in different communities including Indigenous communities, [63]). For example, targeting people’s nostalgia for their own nature experiences may increase their support for environmental initiatives by reminding them of the importance of these experiences for them, their children, and their neighbors. Prior work on significant life experiences suggests that prior exposure to nature is a common trait among those with high environmental awareness and others have pointed to the importance of eco-connections as a pathway to developing climate activism [64,65], but more could be done to study nostalgia and immersion as factors in developing pro-environment attitudes. Further, existing environmental protection campaigns seldom address whether people have any traumatic experiences with nature (floods, fires, etc.), as some participants mentioned in these interviews. Helping people overcome their nature-based trauma may help overcome any resistance to pro-environmental policies. Recognizing the multifaceted nature of eco-connections can improve our understanding of the ways that people interact with nature and help us promote more positive attitudes toward the natural world.

Regarding interventions, our findings align with recommendations to involve youth in “big nature” as described by Kahn and Weiss [36]. Eco-connections are important psychosocial strengths that could be better integrated into developmental and positive psychology literature and perhaps offer pathways to healing for people who have had adverse experiences. For example, the emerging literature on shinrin-yoku (“forest bathing”) suggests that time spent outside can promote wellbeing [66] and creating positive connections with the environment may be helpful as well. Evidence from the study of children and youth’s experiences in nature schools has identified how immersion in greenspace can promote stress reduction, positive risk-taking, and positive mental health. The results also suggest avenues for promoting youth development and strengthening environmental policy communication. Participants gained lifelong lessons and character traits such as responsibility through nature. It could be beneficial for more public schools, not just nature-focused schools, to implement nature regular immersion experiences such as gardening [67], or even more basic experiences such as digging in the ground or leaning on a tree [36].

In conclusion, immersive nature experiences came to be represented nostalgically as among the “happiest times” in peoples’ reminiscences of their childhoods. Time spent in nature helped mark the passage of time and nurtured connections to family members, community members, and animals. Thus, rather than a static backdrop to our lives, this study on the multifaceted dimensions of eco-connections, helps contextualize the ways people’s relationship to and experiences with nature influences people’s lived experiences and potentially facilitates the development of critical developmental processes. For example, participants described how their connections to the rhythm of nature contributed to greater community connectedness as neighbors came together to gather seasonally or enjoy the bounty of home gardens. Just as place connections have been theorized to explain youth’s identify construction [68], future research should explore how eco-connections can function as previously unexamined protective factors and mechanisms to promote community connectedness, healthy development, and resiliency across the life course.

## Data Availability

Data are available from the authors upon reasonable request.
